# The Story of the Insane from Year to Year

**Published:** 1900-11-17

**Authors:** 


### THE STORY OF THE INSANE FROM YEAR TO YEAR.

(Twelfth Series.)

### THE MONMOUTH COUNTY ASYLUM AT ABERGAVENNY.

At this asylum the numbers increased from 1,012 to 1,050. There were 209 first admissions; 30 re-admissions; 74 recoveries: 13 discharged relieved; and 111 deaths. The recoveries stand at 31·4 per cent. on the admissions, and the deaths at 10·7 on the average number resident. Of the deaths 48 were caused by cerebral or spinal diseases; 10 by phthisis; 6 by bronchitis; and 3 by pneumonia. Of the year's admissions 22 owed their insanity to various moral causes 28 to intemperance in drink; 36 to hereditary influence; and 20 were congenital. Dr. Glendinning has a paragraph in his report which well illustrates the extreme importance of early treatment in mental diseases. Four of the recoveries, he says, occurred in patients who had been under one month in the asylum; "25 under three months; 22 under six months; 12 under nine months; and 5 under twelve months;" and "33 of the recoveries were suffering from a first attack of insanity of less than three months' duration on admission." The medical officers give lectures to the staff on subjects connected with the nursing of mental diseases. Twenty-five of the staff entered for the examination of the Medico-psychological Association, and 22 obtained the certificate. This is an admirable beginning, and we hope to hear that Dr. Glendinning has begun a three years' course of instruction for his nurses. The Committee record their "great appreciation of the excellent work being done by Dr. Glendinning in the superintendence of the Institution." The weekly charge to the unions is 7s. 7d. per head. There are 78 private patients, and these pay from 12s. to 30s. a week.

### THE OMAGH DISTRICT ASYLUM.

The limits of accommodation are stated to be for 632 patients, and on January 1st 597 were in residence, the number being increased to 620 on the last day of December. There were 127 first admissions; 44 re-admissions; 73 recoveries; 8 discharged relieved; and 67 deaths. The recoveries were 42·6 per cent. calculated on the admissions; and the deaths were 11 per cent. on the average number resident. Of the deaths 8 were caused by cerebral and spinal diseases; 17 by consumption; 10 by pneumonia, and 3 are returned as having been caused by fever and erysipelas. Moral causes account for the insanity in 19 of the year's admissions; intemperance in drink in 18; congenital defect in 3, and hereditary influence in 27. The asylum was being added to, and when the additions are finished there will be accommodation for 711 patients. Lectures have been given to the attendants during the year, and six have obtained the certificate of the Medico-psychological Association. We are pleased to observe that the committee have increased the wages of these attendants and nurses, and we think if this practice were universal it would give a considerable impetus to trained nursing in asylums. Four of the staff received superannuation allowances during 1898. The annual cost per head was £24 13s. 7d.

### THE SLIGO DISTRICT ASYLUM.

The limits of accommodation are given at 470 beds; but in spite of that there were 569 patients in residence at the beginning of 1898 and 614 at the end of it. There were 109 first admissions, 43 re-admissions, 35 recoveries, 25 discharged relieved, and 41 deaths. The recoveries stand at 23 per cent. of the admissions, and the deaths at 6·9 per cent. of the average number. Both of these are considerably under the average in English asylums. Of the deaths 10 were caused by cerebral and spinal diseases, 8 by consumption, and 5 by pneumonia. Moral causes are believed to have been the cause of the insanity in 17 of the admissions, drink in 4, and hereditary influence in 55. Typhoid fever appeared during the year and caused one death. Four of the other deaths were due to a low form of continued fever, which Dr. Petit seems to think was due to the crowded state of the asylum. Additions are being made to remedy this defect.

### THE WARNEFORD ASYLUM, OXFORD.

There were 90 patients in residence on January 1st, 1898, and 93 on December 31st. During the year 9 patients were admitted: 4 were discharged and 2 died. Like the Nottingham Hospital, this asylum does real charitable work. We notice that 26 of the patients paid 10s. a week or under; 21 paid 15s. a week or under; and 23 paid 25s. a week or under. The average weekly cost was a little under 30s. a week. This sum includes rates, taxes, and repairs; and 13 patients were sent to the Isle of Wight for a month in summer. The Commissioners say that "the hospital was in good order throughout." Neither restraint nor seclusion was employed, and we believe the patients enjoy much freedom.

### THE COPPICE LUNATIC HOSPITAL AT NOTTINGHAM

This asylum contained 94 patients on January 1st, 1898, and on December 31st the number had risen to 98. There were 23 admissions, 7 recoveries, 4 discharged relieved, and 7 deaths. The recoveries were 30·3 per cent. of the admissions, and the deaths were 7·23 per cent. of the average number resident. This is one of the lunatic hospitals that really discharges the duties for which it was called into existence. As the prospectus states, "it is not a private speculation, but was established many years ago for benevolent objects and not pecuniary gain." The ordinary terms are £2 a week, but the committee frequently reduce this payment; and we learn from the table on page 25 of the report that 6 patients are paying from 10s. to 15s. a week; 17 from 15s. to 30s.; 17 pay exactly 30s., and 56 pay the full sum. The Coppice is situated about two miles from the Nottingham station. It is a handsome building, and contains everything essential for the successful treatment of the insane. "The committee express their entire satisfaction with the manner in which Dr. Tate has cared for the welfare of the patients and of the institution."

